# Access to modern methods of contraception in Italy: Will the COVID-19 pandemic be aggravating the issue?

**DOI:** 10.7189/jogh.10.020320

**Published:** 2020-12

**Authors:** Saverio Bellizzi, Giuseppe Pichierri, Catello Mario Panu Napodano, Sara Picchi, Serena Fiorletta, Maria Grazia Panunzi, Edoardo Rubattu, Alessandra Nivoli, Liliana Lorettu, Antonella Amadori, Susanna Padrini, Anna Rita Ronzoni

**Affiliations:** 1 Medical Epidemiologist, Independent consultant, Geneva, Switzerland; 2 Kingston Hospital NHS Foundation Trust, Microbiology Unit, Kingston Upon Thames, United Kingdom; 3 Infectious Diseases Department, AOU Sassari, University of Sassari, Sassari, Italy; 4 Associazione Italiana Donne per lo Sviluppo (AIDOS); 5 University of Sassari, Sassari, Italy; 6 ATS Sardegna UONPIA, Dipartimento Salute Mentale Area Nord, Olbia, Italy; 7 Italian Association for Solidarity among People (AISPO), Port Said, Egypt; 8 Eastern Mediterranean Region Office for the World Health Organization, Cairo, Egypt

The strain on health system imposed by the current COVID-19 pandemic is undoubtedly impacting the sexual and reproductive health of women and girls of many countries in the world. Reconfiguration of health systems to cope with the epidemic has led to the forced closure of health services deemed not essential as well as diversion of health care workers to fulfill other needs.

In addition, physical distancing and travel bans are having adverse effects on access to and supply of contraceptive commodities. Manufacturing of specific pharmaceutical components is, for instance, causing a shortage of condoms [1].

A mathematical model exercise done by the Guttmacher Institute has estimated a 10% proportional decline in the use of short- and long-acting reversible contraceptive methods in low- and middle-income countries (LMICs) due to reduced access [2]. Other pieces of very recent research have predicted much worse scenarios, with decline in sexual and reproductive health services up to 80% [3].

Access to contraception is not just an LMICs issue: the Contraception Atlas 2019 initiative, powered by the European Parliament Forum for Sexual and Reproductive Rights (EPF) in partnership with a group of experts in sexual and reproductive health and rights, showed that for many European countries, ensuring that people have choice over their reproductive lives is not a priority [4].

Specifically, the Atlas tracks government policies on access to contraceptive methods, family planning counselling and the provision of online information on contraception in 46 European states. In the 2019 European Contraception Atlas, Italy ranked just 26th out of the total 46 countries.

## THE ITALIAN CONTEXT

In order to analyze the bad performance of Italy in terms of access to contraception, a national ATLAS has been promoted in order to understand also the impact of the regional differences. Despite the fact that access to contraception is regulated at the national level, in Italy the organization and implementation of contraceptive services are left to the regional governments. For this reason, only 4 regions out of 21 have introduced the provision of free contraception [5].

The 2019 Italian Contraception Atlas indicates two main results which have to be considered when the effect of COVID 19 on contraception is taken into account in terms of advocacy and emergency policies. The first is the huge differences among regions with the historical divide between North and South (with the only exception of Puglia which is the first region that has introduced free contraception at regional level). Such regional differences are multi-factorial and likely driven by cultural and social aspects. The second is a widespread tendency to fund programs which handle the access to the information of contraceptive methods more than those directed to their provision. This is as a result of the scarcity of public resources as well as the strict manner in which related budgets can be spent. Information programs are preferentially funded as they are less expensive than providing actual contraceptive methods. The fragmentation of the policies has an impact on the equal opportunity to have free access to contraception in Italy. There is evidence that a lack of general direction at national level generates gaps among territories which could be addressed with several initiatives, as the introduction of a sort of minimum level of assistance and the vocational training of medical professionals, thus facilitating integrated programmes on contraception and reproductive health, or by improving what has been already implemented as the web page on the portal of the Ministry of Health dedicated to contraception.

Despite the establishment of the counseling centers under the Law n. 405 in 1975 [6], with the aim of supporting “responsible parenthood” through providing information on contraception and by protecting the health of women and their children, institutionalized provision of appropriate information on contraception is still broadly lacking: 89% of boys and 84% of girls seek for information related to sexual and reproductive health in the internet [7]. On the other hand, 68% of boys and 76% of girls have never been in a counseling center [7].

In Italy, family planning centers are often under-resourced and understaffed; as a consequence, individual counselling is rarely provided by interdisciplinary teams, which might affect the quality of the service depending on users’ needs [8]. Moreover, few centers are directly linked to hospitals or specialized services, so that women are not always referred to specialists when appropriate. Importantly, family planning centers are not easily accessible outside the larger cities [8].

**Figure:**
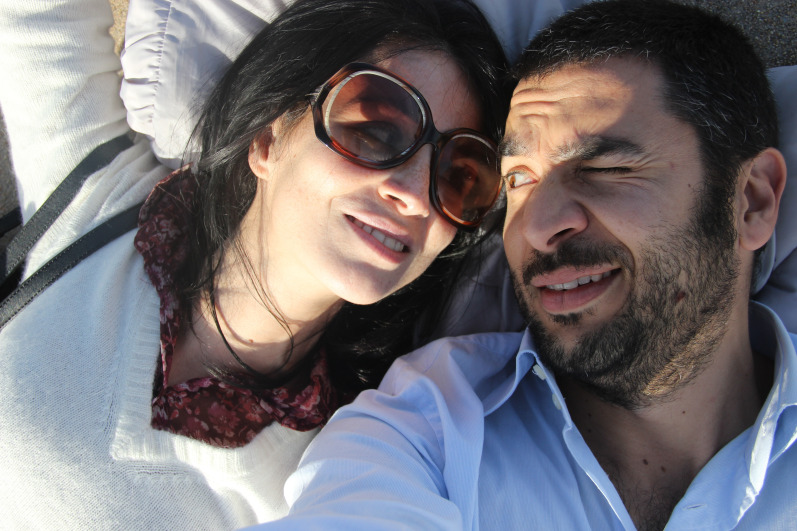
Photo: A young Italian couple engaging in discussion about contraception (from Saverio Bellizzi’s collection, used with permission).

In the North-Eastern regions plus Lombardy and Molise there is less than 1 family planning center per 10 000 women. This picture is worse even compared to Southern regions (where the average is 1 to 2) as well as to other regions in the North and the Center (where the average is 2 to 3) [8].

As far as the use of contraception is concerned, 26% of adolescents use *withdrawal* as preferred method of contraception while 11% relies on *standard days* and 10% do not use any contraceptive method [7].

This non-optimal situation is compounded by the largely stigmatizing vision on reproductive health, specifically around abortion and some modern methods of contraception like the pill. In fact in Italy, despite the legalization of voluntary interruption of pregnancy under the Law n. 194 in 1978 [9], conscientious objection remains one of the main obstacles, with a percentage of 68.4% of gynecologists who avail themselves of this option [10], preventing women in Italy from accessing the human right to have control over their bodies and the legal right to terminate their pregnancies, often forcing them to seek treatment abroad.

Financial accessibility represents another key bottleneck: the intrauterine device (IUD), a very effective contraceptive method with a long-term effect on avoiding or delaying pregnancy, could be as costly as 200 euros, which is well beyond the reach of all women and girls belonging to vulnerable social group who cannot afford this method. As for the pill, the Italian Pharmaceutical Agency has classified in the “C” category, which makes it almost completely in charge of women and requires prescription.

## ATLAS REPORT RECOMMENDATIONS

One of the main recommendations of the 2019 Atlas Report refers to the need for financially strengthening counseling centres, which should be much more widespread in the territory (1 every 20 000 inhabitants instead of the current 1 every 35 000). The counseling centres, perform key function in providing information, support and promotion of women's health. Data collected during an ISS (Istituto Superiore di Sanita’) survey carried between 2018-2019, showed that more than 75% of the counseling centres involved in the survey provide support and care related to sexuality, contraception, VIP’s pathway, preconceptional health, family planning, birth pathway, sexually transmitted diseases, cancer screening, menopause and postmenopause [11]. Furthemore, 25% of the overall counseling centres in Italy offer free contraceptives [11]. Sexual education in the schools is another crucial recommendation.

## CONCLUSIONS

Despite the disruption due to the current COVID-19 pandemic, it is essential to ensure that women can have control over their bodies and their sexual and reproductive life, and protect the access to modern contraceptives and family planning services for both men and women.

In line with the call made by the UN secretary general, access to contraception must be streamlined, even without prescription and free of charge. When regular health care services are disrupted, access to long-acting and emergency contraceptives becomes even more essential.
